# Extended Indications for Sleeve Lobectomy: A Single‐Center Experience of Surgical Management of Central Pulmonary Metastases

**DOI:** 10.1111/1759-7714.70294

**Published:** 2026-04-30

**Authors:** V. Verzeletti, A. Busetto, A. Bonis, F. Shamshoum, G. Pagliarini, M. Mammana, E. Faccioli, A. Rebusso, G. Cannone, G. M. Comacchio, M. Schiavon, A. Dell’ Amore

**Affiliations:** ^1^ Department of Cardiac, Thoracic, Vascular Sciences and Public Health University of Padua Padua Italy; ^2^ Thoracic Surgery Unit and Lung Transplant Centre, Division of Surgery University ‐Hospital of Padua Padua Italy

**Keywords:** central pulmonary metastasis, lung metastasis, quality of life, sleeve lobectomy, surgery

## Abstract

**Background:**

The role of bronchial sleeve resection for centrally located pulmonary metastases remains poorly defined, as surgery in metastatic disease is often perceived as excessively aggressive. However, in selected patients, this parenchyma‐sparing technique may offer durable local control and significant symptomatic relief. This study reports a single‐center experience with sleeve resections performed for metastatic, centrally located pulmonary lesions.

**Methods:**

All consecutive patients undergoing bronchial sleeve resection for metastatic disease at Padua University Hospital between January 2000 and August 2025 were retrospectively reviewed. Clinical characteristics, operative details, perioperative outcomes, and follow‐up data were collected. Patients treated with sleeve resections for primary lung cancer were excluded.

**Results:**

Eighteen patients were included. Most had good performance status, and 66% received preoperative systemic therapy. Single sleeve resections were performed in 72% and double sleeves in 28%. Surgical access was thoracotomy in 72% and VATS in 28%. No in‐hospital, 30‐day, or 90‐day mortality occurred. Postoperative symptom resolution was achieved in 94% of patients. The most frequent histology was colorectal adenocarcinoma. Median follow‐up was 26 months, with a median disease‐free survival of 22 months. Local recurrence occurred in only one case, and no bronchial stump recurrences were observed.

**Conclusion:**

Bronchial sleeve resection for centrally located pulmonary metastases can be feasible and safe in a carefully selected subset of patients. It provides effective restoration of airway patency, good local control, and acceptable long‐term outcomes. Larger multicenter studies are needed to further clarify its role within multidisciplinary management.

## Graphical abstract

Sleeve resection in the metastatic setting represents a valid therapeutic option for selected patients.

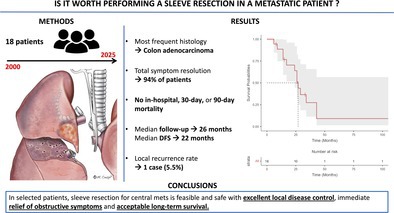

## 1 Introduction

In recent years, the surgical management of centrally located pulmonary lesions has evolved significantly, particularly in the setting of metastatic disease [1]. Among the surgical options, bronchial sleeve resection stands out as a technically demanding but parenchyma‐sparing procedure traditionally used for centrally located primary lung tumors [2]. However, its role in patients with centrally located metastatic disease remains controversial and poorly defined [3].

Performing a bronchial sleeve resection in a patient with metastatic cancer may be perceived as overly aggressive, given the technical complexity of the procedure and the systemic nature of metastatic disease [4, 5]. In this context, many clinicians may question whether the potential benefits of surgery justify the risks and resources required, especially when compared to less invasive local treatments [6]. Most of these patients are typically managed with loco‐regional therapies, such as external beam radiotherapy, brachytherapy, or endoscopic interventions including tumor debulking or stent placement. These approaches aim to relieve symptoms and maintain airway patency, often with acceptable results in terms of quality of life and short‐term disease control [7].

However, in a carefully selected subset of patients, surgical resection may offer additional benefits. In cases where the metastatic lesion is isolated, centrally located, and causing significant symptoms or airway obstruction, bronchial sleeve resection could serve as a definitive treatment—either to control local disease or to improve the patient’s functional status and quality of life [8]. Advances in perioperative care and surgical techniques have made complex resections more feasible and safer, even in oncologically challenging scenarios [9].

While surgery has long been considered a last resort or even contraindicated in metastatic patients, emerging evidence suggests that under specific circumstances, resection may contribute meaningfully to multidisciplinary cancer care. Particularly in patients with long disease‐free intervals, good performance status, and limited metastatic burden, surgical intervention might not only alleviate symptoms but potentially impact overall survival.

In this study, we present our single‐center experience with bronchial sleeve resections performed for centrally located metastatic lesions.

## 2 Materials and Methods

This study includes all consecutive patients who underwent bronchial sleeve resection for metastatic reasons at the Thoracic Surgery Unit of Padua University Hospital, from January 2000 to August 2025. Data were collected from a prospectively maintained retrospective database including demographic information, history of prior treatments, operative reports, and information about the postoperative course, including follow‐up controls after hospital discharge. Patients who received sleeve resections for primary lung cancers were excluded from this analysis.

This study complies with the ethical standards of the institutional research committee and with the 1964 Helsinki declaration and its later amendments. All patients signed an informed consent form for the use of anonymized data for research purposes.

## 3 Results

### 3.1 Baseline Characteristics of the Population

Patients’ baseline features are summarized in Table 1.

**TABLE 1:** General features of the population.

	*N* = 18
Gender	
Male	8 (44%)
Female	10 (56%)
Age	64 (58, 70)
BMI	27.3 (24.4, 29.0)
Smoking	
Yes	2 (11%)
No	8 (44%)
Former	8 (44%)
Pack/Years	4 (0, 31)
FEV1 (L/min)	1.90 (1.67, 2.74)
Charlson Comorbidity Index	
≤ 5	5 (28%)
> 5	13 (72%)
ECOG Performance Status	
0	13 (72%)
1	5 (18%)
Systemic therapy before surgery	
No	6 (44%)
Yes	12 (66%)
Previous Omolateral Surgery	
No	16 (89%)
Yes	2 (11%)

*Note:* *n* (%); Median (IQR).

A total of 18 patients were included in the study. The median age was 64 years (IQR: 58–70), and the median BMI was 27.3 kg/m^2^ (IQR: 24.4–29.0). Ten patients (56%) were female and eight (44%) were male. Regarding smoking status, 2 patients (11%) were current smokers, 8 (44%) were non‐smokers, and 8 (44%) were former smokers. The most frequent histology of the primary tumor was colorectal adenocarcinoma (8 patients, 39%), followed by clear cell renal carcinoma (3 patients, 17%), uterine adenocarcinoma (2 patients, 6%), skin melanoma (2 patients, 6%), laryngeal squamous cell carcinoma (1 patient, 6%), breast adenocarcinoma (1 patient, 6%), and fibrosarcoma (1 patient, 6%).

The median preoperative FEV1 was 1.90 L/min (IQR: 1.67–2.74). The Charlson Comorbidity Index was > 5 in 13 patients (72%). Most patients (72%) had an ECOG performance status of 0, while 5 patients (18%) had a status of 1. Twelve patients (66%) received systemic therapy before surgery. Two patients (11%) had undergone previous ipsilateral surgery.

### 3.2 Intraoperative and Postoperative Data of the Patients

Intraoperative and postoperative data of the patients are shown in Table 2.

**TABLE 2:** Intraoperative and postoperative data of the 18 patients.

Type of sleeve lobectomy	
Right upper	8 (44%)
Left upper	6 (33%)
Middle	1 (6%)
Left lower	1 (6%)
Bilobectomy	2 (11%)
Additional vascular sleeve resection	
No	13 (72%)
Yes	5 (28%)
Surgical access	
Thoracotomy	13 (72%)
VATS	5 (18%)
Surgery time (min)	238 (170, 302)
Estimated blood loss (ml)	
≤ 150	13 (72%)
> 150	5 (18%)
Complications	
Minor	18 (100%)
Major	0 (0%)
Clavien Dindo classification	
0	11 (61%)
1	1 (5.6%)
2	4 (22%)
3a	2 (11%)
Blood Patch	1
Chest Tube	1
Drainage removal (days)	
≤ 2	1 (5.6%)
> 2 ≤ 5	5 (28%)
> 5 < 10	9 (50%)
≥ 10	3 (17%)
In‐hospital lenght of stay (days)	
≤ 3	1 (5.6%)
> 3 ≤ 7	5 (28%)
> 7 ≤ 10	9 (50%)
> 10	3 (17%)
In‐hospital mortality	
None	18 (100%)
30‐day mortality	
None	18 (100%)
90‐day mortality	
None	18 (100%)

*Note:* *n* (%); Median (IQR).

Right upper sleeve lobectomy was performed in 8 patients (44%), left upper in 6 (33%), middle lobectomy in 1 (6%), and left lower lobectomy in 1 (6%). Two patients (11%) underwent bilobectomy. A vascular sleeve lobectomy was also performed in 5 patients (28%).

Thoracotomy was the surgical access for 13 patients (72%), while VATS was chosen in 5 patients (28%). The median surgical time was 238 min (IQR: 170–302). Estimated blood loss was ≤ 150 mL in 13 patients (72%) and > 150 mL in 5 patients (28%).

No major complications were observed. According to the Clavien‐Dindo classification, 11 patients (61%) had no postoperative complication (grade 0), 1 (5.6%) had grade I, 4 (22%) had grade II, and 2 (11%) had grade IIIa (1 blood patch, 1 additional chest tube placement). Pleural drainage was removed within 2 days in 1 patient (5.6%), between 3 and 5 days in 5 patients (28%), between 6 and 9 days in 9 patients (50%), and after 10 days in 3 patients (17%). Length of in‐hospital stay was ≤ 3 days in 1 patient (5.6%), between 4 and 7 days in 5 patients (28%), and longer than 7 days in 13 patients (72%).

No cases of in‐hospital, 30‐day, or 90‐day mortality were observed.

### 3.3 Histological Data and Follow‐Up of the Patients

**FIGURE 1:**
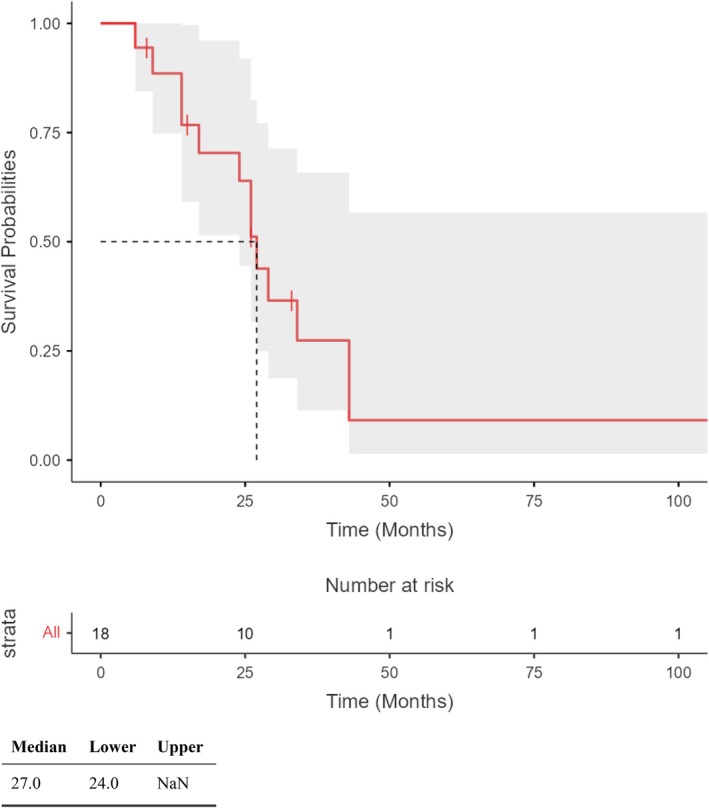
Overall survival.

**FIGURE 2:**
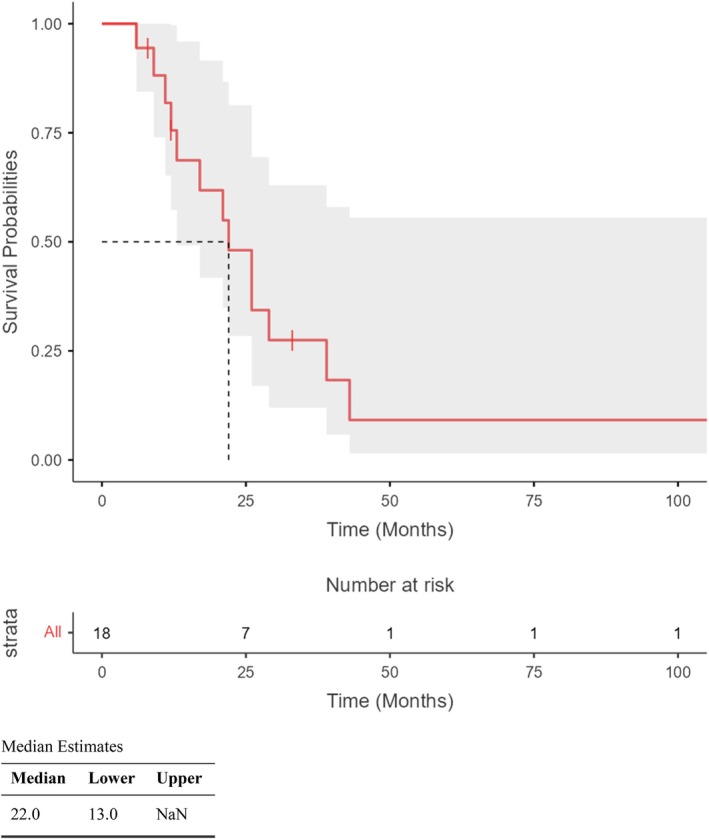
Diesase free survival.

Data about patients follow‐up and final histological examination are shown in Table 3, Figures 1 and 2.

**TABLE 3:** Pathological data and follow‐up of the patients.

Tumor size (cm)	3.50 (2.85, 4.15)
Histology	
Colo‐rectal adenocarcinoma	8 (39%)
Clear cell renal carcinoma	3 (17%)
Uterine adenocarcinoma	2 (6%)
Skin melanoma	2 (6%)
Laringeal squamous cell carcinoma	1 (6%)
Breast adenocarcinoma	1 (6%)
Fibrosarcoma	1 (6%)
Adjuvant therapy	
No	12 (78%)
Yes	6 (22%)
Type of adjuvant therapy	
Chemotherapy	3 (60%)
Radiotherapy	3 (40%)
Type of late complications	
Bronchial Stenosis	1 (6%)
Treatment of late complications	
Endoscopic stent	1 (6%)
Recurrence	
Distance	5 (83%)
Local	1 (17%)
Type of local recurrence	
Loco‐regional	1 (6%)
Distance	6 (44%)
Treatment of local recurrence	
Radiotherapy	1
Follow‐up (months)	26 (14, 32)
Status	
Alive	5 (28%)
Dead	13 (72%)

*Note:* *n* (%); Median (IQR).

The median tumor size was 3.50 cm (IQR: 2.85–4.15). Adjuvant therapy was administered to 6 patients (22%), including chemotherapy in 3 (60%) and radiotherapy in 3 (40%).

Late airway complications occurred in 1 patient (6%), who developed bronchial stenosis and was treated with endoscopic stent placement 1 year after surgery.

The median follow‐up length was 26 months (IQR: 14–32). At the end of follow‐up, 5 patients (28%) were alive and 13 (72%) died. The median disease free survival (DFS) was 22 months. Tumor recurrence was observed in 6 patients (33%), with extrathoracic recurrence in 5 cases and local recurrence in 1 patient. No bronchial stump recurrence was observed. The single case of local recurrence was treated with radiotherapy.

## 4 Discussion

This case series testifies to the role of bronchial sleeve resections in a specific subset of patients with centrally located pulmonary metastases from extrathoracic primary tumors.

The selection of patients is of primary relevance, considering that a complex surgical approach in a metastatic setting could be interpreted as an out‐of‐guidelines treatment. Therefore, there are both pathological and clinical reasons to justify such an invasive treatment when particular conditions exist.

First of all, we must consider the patient’s overall condition, as they must obviously be fit for major lung surgery. Our experience with these patients has taught us to contextualize lung reserve and respiratory function tests since many of them have collapsed parenchymal areas due to the central tumor that do not contribute to ventilation even before surgery.

Moreover, the pathological diagnosis of the primary tumor is pivotal for patient selection, as certain cancers exhibit indolent metastatic behavior. Consequently, management may shift from aggressive combined local and systemic treatments to prolonged surveillance with targeted local therapies aimed at controlling recurrent disease [10]. Consistent with previous reports on central pulmonary metastases, the most common histologies undergoing surgical resection in our series were colorectal adenocarcinoma, renal cell carcinoma, breast cancer, and gynecologic malignancies [11]. Notably, these tumors share a tendency to present as isolated, slow‐growing pulmonary metastases, sometimes years after initial treatment [12].

Beyond the indolent natural history of these tumors, our intraoperative experience showed interesting results. Despite sleeve lobectomies being a technically demanding surgery, our data demonstrate a favorable perioperative profile: no in‐hospital, 30‐day, or 90‐day mortality, and no major complications (Clavien‐Dindo > IIIb). Most complications were minor (Grade I–II) and manageable without invasive intervention. These outcomes compare favorably with historical data on pulmonary metastasectomy, particularly considering that broncho‐vascular sleeve resections—associated with greater technical complexity and risk—were performed in 28% of our patients. These findings support that, in high‐volume centers, complex airway reconstructions can be safely undertaken even in the rare setting of metastatic disease.

Finally, a noteworthy aspect is certainly the effectiveness of sleeve resection in restoring airway patency and resolving symptoms which cannot be overstated. The local growth of central metastases often causes severe bronchial obstruction, leading to dyspnea, cough, recurrent infections, and compromised lung function [13]. In addition, conventional endoscopic management of these ab‐extrinseco obstructive conditions is not feasible, and stenting should not always be considered as a durable treatment. Moreover, dislocation, migration, or pressure‐related complications (mucosal erosion or bronchial perforation) may represent a concrete threat for the patient. Several studies have reported limited outcomes with endoscopic debulking for renal metastases involving the central airways. One such study demonstrated that more than 50% of patients required multiple interventions, with median survival durations significantly lower than those observed in our surgical cohort [14]. While endoscopic procedures may offer temporary palliation, they do not achieve radical excision or definitive local control. Conversely, surgical sleeve resection achieves both symptomatic relief and oncologic control, standing as a durable and resilient solution in slowly growing metastatic cases. Considering surgery, in our series, 17 out of 18 patients (94%) postoperatively experienced complete regression of obstructive respiratory symptoms. Only one patient developed late bronchial stenosis at the anastomotic site, which was unrelated to tumor recurrence and was successfully and durably managed by endoscopic stent placement. Furthermore, no bronchial stump recurrences were observed during follow‐up, suggesting effective and complete local disease control, rather than the merely palliative intent. This study has several limitations that should be acknowledged when interpreting the findings. First, its retrospective design inherently introduces risks of selection bias and incomplete data capture. Second, this is a single‐center experience from a high‐volume thoracic surgery unit with specific expertise in complex airway and broncho‐vascular reconstructions. As such, the results may not be generalizable to centers with lower case volumes or limited experience in sleeve procedures. The favorable perioperative outcomes observed in our cohort likely reflect both careful patient selection and the technical proficiency of the surgical team. Moreover, the sample size is small, reflecting the rarity of centrally located pulmonary metastases amenable to sleeve resection. With only 18 patients included over a 25‐year period, the study is underpowered to detect subtle differences in survival, recurrence patterns, or complication rates across subgroups. Finally, the study lacks a control group of patients treated with alternative approaches, such as endoscopic debulking, stenting, or non‐sleeve pulmonary resections. As a result, the relative benefit of sleeve resection over other local treatments cannot be definitively established. Despite these limitations, this study provides meaningful preliminary evidence supporting the feasibility, safety, and potential oncologic value of bronchial sleeve resection in carefully selected patients with centrally located pulmonary metastases. It highlights a niche but relevant role for complex airway surgery within a multidisciplinary metastatic disease framework and underscores the need for larger, multicenter studies to validate these findings.

## 5 Conclusion

Our findings suggest that in a well‐selected subset of patients, bronchial sleeve resection for central metastatic lesions is both feasible and safe, providing excellent local disease control, immediate relief of obstructive symptoms, and acceptable long‐term survival. Future multicentric studies with larger cohorts and comparative analyses are warranted to further define the role of surgery in this unique clinical context.

## Author Contributions

**V. Verzeletti:** conceptualization, investigation, writing – original draft, methodology, validation, software, formal analysis, project administration, data curation. **A. Busetto:** conceptualization, investigation, writing – original draft, validation, methodology, formal analysis, software, project administration, data curation. **A. Bonis:** conceptualization, methodology, formal analysis, writing – review and editing. **F. Shamshoum:** data curation, software, formal analysis. **G. Pagliarini:** data curation, software, formal analysis. **M. Mammana:** writing – review and editing, supervision, visualization. **E. Faccioli:** visualization, writing – review and editing, supervision. **A. Rebusso:** validation, visualization, supervision. **G. Cannone:** validation, visualization, supervision. **G. M. Comacchio:** methodology, validation, writing – review and editing, supervision. **M. Schiavon:** validation, visualization, supervision. **A. Dell’ Amore:** supervision, writing – review and editing, project administration.

## Funding

The authors have nothing to report.

## Ethics Statement

The study was conducted in accordance with the Declaration of Helsinki.

## Consent

Written informed consent has been obtained from the patients to publish this paper.

## Conflicts of Interest

The authors declare no conflicts of interest.

## Data Availability

Other patients’ information is available from the corresponding author on reasonable request.
